# English Speakers’ Perception of Non-native Vowel Contrasts in Adverse Listening Conditions: A Discrimination Study on the German Front Rounded Vowels /y/ and /ø/

**DOI:** 10.1177/00238309241254350

**Published:** 2024-06-10

**Authors:** Stephanie Kaucke, Marcel Schlechtweg

**Affiliations:** Institute for English and American Studies, Carl von Ossietzky Universität Oldenburg, Germany; Cluster of Excellence “Hearing4All,” Germany

**Keywords:** Non-native speech perception, vowels, speech in noise, discrimination task

## Abstract

Previous research has shown that it is difficult for English speakers to distinguish the front rounded vowels /y/ and /ø/ from the back rounded vowels /u/ and /o/. In this study, we examine the effect of noise on this perceptual difficulty. In an Oddity Discrimination Task, English speakers without any knowledge of German were asked to discriminate between German-sounding pseudowords varying in the vowel both in quiet and in white noise at two signal-to-noise ratios (8 and 0 dB). In test trials, vowels of the same height were contrasted with each other, whereas a contrast with /a/ served as a control trial. Results revealed that a contrast with /a/ remained stable in every listening condition for both high and mid vowels. When contrasting vowels of the same height, however, there was a perceptual shift along the F2 dimension as the noise level increased. Although the /ø/-/o/ and particularly /y/-/u/ contrasts were the most difficult in quiet, accuracy on /i/-/y/ and /e/-/ø/ trials decreased immensely when the speech signal was masked. The German control group showed the same pattern, albeit less severe than the non-native group, suggesting that even in low-level tasks with pseudowords, there is a native advantage in speech perception in noise.

## 1 Introduction

Our native language significantly affects how we perceive non-native speech sounds. Although infants are able to discriminate all kinds of speech sounds, they lose this ability during their first year of life as they specialize in the sound system of the language that they are acquiring (Werker & Lalonde, 1988; Werker & Tees; 1984). Due to this perceptual reorganization in infancy, adults typically have difficulties in discriminating sounds that do not occur in their native language as they assimilate them to their native categories (e.g., Flege, 1995). For instance, it has been shown that Japanese speakers have difficulties in discriminating between English /r/ and /l/ (Bradlow et al., 1999), that Spanish speakers struggle to distinguish tense from lax vowels (Cebrian et al., 2021), and that Korean speakers cannot differentiate voiced from voiceless stops (Kazanina et al., 2006). Despite the fact that we have by now a good understanding of how non-native speakers deal with unknown sounds, most studies have only concentrated on quiet listening conditions. However, in everyday life, speech perception rarely takes place in quiet as we are almost always surrounded by some kind of background noise, which represents an additional perceptual difficulty for non-native speakers. This study thus investigates English speakers’ perception of the German front rounded vowels /y/ and /ø/ both in quiet and in one type of noise, namely white noise.

### 1.1 English listeners’ perception of front rounded vowels in quiet

Front rounded vowels do not occur in English and hence pose a problem for native English listeners. It has been shown that they perceptually assimilate these vowels to the categories of back rounded vowels (e.g., Levy, 2009; Levy & Strange, 2008; Strange et al., 2005). For instance, in a perceptual assimilation task, Strange et al. (2005) found that 97% of the German front rounded vowels were categorized as back vowels: /y/ was consistently perceived as most similar to /u/, and /ø/ was assimilated to /u/, /ʊ/, or /oʊ/, with no clear preference for either of these back vowels. Because there was no single category that /ø/ could be mapped on, Strange et al. regarded /ø/ as uncategorizable for American English listeners. Furthermore, Kingston (2003) found that American English listeners were much better at discriminating the German mid vowel contrast of /o/ and /ø/ than the high vowel contrast of /u/ and /y/.

Interestingly, even listeners with extensive knowledge of a language in which the front rounded vowels occur phonemically (such as German or French) treat the front rounded vowels and the back rounded vowels as a single category (Darcy et al., 2012; Levy & Strange, 2008; Melnik-Leroy et al., 2021). For instance, Darcy et al. (2012) examined the phonological representations of the /y/-/u/ and /œ/-/ɔ/ contrasts for learners of French, who were native speakers of English. Although advanced learners had established individual phonological categories for the two non-native front rounded vowels, intermediate learners treated words with /y/ and /u/ (but not /œ/ and /ɔ/) as homophones. However, despite having established these distinct phonological categories, advanced learners still struggled to differentiate /y/ from /u/ in an ABX discrimination task. Furthermore, Levy (2009) also found that learners of French who were native speakers of American English robustly assimilated French /y/ and /œ/ to English back vowel categories, but this effect was stronger in alveolar than in labial contexts. To understand this context-dependent effect in such a phonological assimilation, we need to consider the acoustics of English vowels. Although English does not have front rounded vowels, in alveolar contexts, the back rounded vowels become fronted and thus have higher F2 values (Strange et al., 2007). This has been reported for several varieties of English, such as American (Strange et al., 2005), British (Hawkins & Midgley, 2005), and Australian English (Cox & Palethorpe, 2001). Fronting high back rounded vowels is frequently observed in the world’s languages. In a coronal context such as /tut/, the tongue body has to move forward before and after the vowel, which results in higher values of the second formant (Flemming, 2003; Harrington et al., 2011).

Although the English vowel inventory leaves room for this type of allophonic variation, the German one does not because the latter relies on a phonemic distinction between the front unrounded vowel /i/, the front rounded vowel /y/, and the back rounded vowel /u/. This was confirmed by Strange et al. (2007), who showed in an acoustic analysis that German back rounded vowels are articulated further back and are fronted less than the respective sounds in American English. They argue that coarticulation is constrained in a language-specific way. That is, to ensure a successful identification on the side of the listener, speakers must maintain the acoustic distinctiveness between vowels. In German, both front and back vowels move forward to a similar degree to maintain the acoustic difference between them. In American English, on the contrary, the front vowels remain relatively stable regardless of phonetic context, whereas the back vowels vary greatly along the F2 dimension. But if the German front rounded vowels are acoustically much closer to the front unrounded vowels than to the back rounded vowels, as research has shown (Strange et al., 2005, 2007), why do English listeners perceive them as back rounded vowels? Strange et al. (2007) argue that German front rounded vowels are too deviant from the stable English front (unrounded) vowel categories to still be classifiable as another front vowel. Moreover, because English allows back vowels to deviate substantially along the F2 continuum depending on the phonetic context, it seems plausible that native English listeners perceive the front rounded vowels as allophonic variations of back vowels.

Thus, to further examine this perceptual assimilation pattern of front rounded vowels that has been shown for English listeners in languages such as German and French, this study investigates the influence of adverse listening conditions on English listeners’ ability to discriminate German vowels in an Oddity Discrimination Task, with a specific focus on the vowels /y/ and /ø/.

### 1.2 Non-native speech perception in noise

Apart from cross-linguistic variation itself, other aspects can also affect how language users perceive non-native linguistic properties. One case in point is the quality of the input the listener is exposed to. In daily life, adverse listening conditions represent an additional obstacle for all individuals, but especially for non-native speakers. Several studies have shown that non-native speakers are disproportionately affected by noise compared with native speakers (e.g., Broersma & Scharenborg, 2010; Cutler et al., 2004; Jin & Liu, 2014; Mi et al., 2013). As Cutler et al. (2004) argue, this is due to non-native speech perception in noise being slower and less efficient at several levels of processing (such as phonemic, syntactic, and semantic). Yet the vast majority of studies that examined the effect of noise on non-native speech perception so far used English as a target language (e.g., Bent & Bradlow, 2003; Broersma & Scharenborg, 2010; Cutler et al., 2004; Hintz et al., 2022; Jin & Liu, 2014; MacKay et al., 2001; Mi et al., 2013; Rogers et al., 2006; Shimizu et al., 2002). That is, participants were never naïve and had already had extensive knowledge of the language. This study, in contrast, concentrates on (native English) speakers without any prior knowledge of the target language (i.e., German). Experienced learners of German would have had some phonetic knowledge of the target language which they could have used to their advantage, albeit less well than native German speakers. To be able to directly compare a native and a naïve non-native group in their low-level perception of speech sounds, we used pseudowords instead of real German words in this study. This way, neither participant group could use any lexical or semantic information to recover from the disruption in the speech signal.

On a word and sentence level, it has been shown that adverse listening conditions posed more challenges for non-native than native speakers, even if the non-natives were highly proficient (Hintz et al., 2022; Mayo et al., 1997; Rogers et al., 2006). This discrepancy between the two groups was also found in low-level discrimination tasks (Cutler et al., 2004, 2008; García Lecumberri & Cooke, 2006). For instance, García Lecumberri and Cooke (2006) and Cutler et al. (2008) tested Spanish and Dutch speakers, respectively, on their consonant discrimination abilities in English. Both studies found that when an unvarying context such as /aCa/ was given, native speakers were better able to compensate for the degraded speech signal than the non-native speakers. That is, if there is less uncertainty about the identity of the target sound, native speakers are able to exploit even the smallest low-level phonetic cues to compensate for the adverse listening conditions. Non-native speakers, in turn, suffer more because their phonetic knowledge of the language will be less extensive. Cutler et al. (2007) suggest that the reason for the difference between native and non-native speakers’ accurate perception of phonetic cues might be rooted in the relevance of those cues in the listeners’ native language. Thus, non-native listeners rely on fewer or different cues compared with native listeners and cannot yet efficiently use the full array of cues that are relevant for phoneme identification in the non-native language (Cebrian, 2006), especially in noise.

## 2 Methodology

In this study, we used an Oddity Discrimination Task, similar to the one used in Darcy and Krüger (2012), to test English (test group) and German (control group) participants in their ability to discriminate German vowel contrasts in quiet and white noise.

Vowels were either contrasted with other vowels of the same height (high vowels: /iː, yː, uː/; mid vowels: /eː, øː, oː/) or with /aː/ in a control condition. The stimuli consisted of consonant, vowel, and consonant (CVC) items that were pseudowords in both English and German and adhered to the phonotactics of both languages. Hence, both the test group as well as the control group could be tested on the same stimuli.

### 2.1 Participants

In the test group, 46 native speakers of English participated in the experiment (25 women; mean age = 27.9 years, age range = 18–67 years).^1^ None of them had any prior knowledge of German. In the control group, 44 native speakers of German were tested (31 women; mean age = 23.5 years, age range = 18–38 years).^2^ All participants reported normal hearing. Because the study was run online, we could not run an auditory screening and had to rely on participants’ own statements.

### 2.2 Materials

All stimuli were recorded by three female native German speakers with a Tascam DR-100MKIII Linear PCM Recorder in a quiet room.^3^

The two participant groups were tested on the same stimuli. The stimuli were divided into eight sets of CVC quadruples that only differed in the vowel. Half of the quadruples belonged to the high vowel group (/iː, yː, uː/ plus /aː/ as a control) and the other half belonged to the mid vowel group (/eː, øː, oː/ plus /aː/ as a control). Only consonants that occurred both in English and in German were used in the consonant frames and were drawn from the set /p, t, d, k, f, v, m, n/. Only items of the same quadruple set were contrasted with each other in a trial. A list of all stimuli can be found in the Appendix.

Three practice trials preceded the experiment. Here, only vowel height contrasts that were not part of the study were used, such as /iː/ versus /eː/.

To create the stimuli for the noise conditions, we used white noise as a masker that was laid over the speech signal in Praat (Boersma & Weenink, 2023) by means of the Praat extension Vocal Toolkit (Corretge, 2012–2022). Two signal-to-noise ratios (SNRs) were used: 8 and 0 dB. The masking started at the onset of the speech stimulus and ended at its offset. All items were normalized to 70 dB.

We favored energetic rather than informational masking as it allowed us to control for the effect of the added noise across the two participant groups. It has been shown that the effect of informational masking due to multitalker babble noise is dependent on whether the participants’ native language matches the babble language or not (e.g., Van Engen, 2010, 2012; García Lecumberri & Cooke, 2006). Van Engen (2010), for instance, ran a sentence recognition task in English with L1 English-speaking and L1 Mandarin-speaking participants in which she compared the effect of the babble language on both participant groups. Results showed that there was not only a detrimental effect of similarity between target and noise language but also that it was easier to tune out a masker in an unknown language than in a native language. To avoid such an advantage for any group in this study, we decided to mask our stimuli using white noise instead of babble noise in either language.

Most studies that investigated non-native speech perception in noise, however, used speech-shaped noise (e.g., Cutler et al., 2007; García Lecumberri & Cooke, 2006; Hintz et al., 2022; Jin & Liu, 2014; Liu & Jin, 2013; Mi et al., 2013) or multitalker babble noise (e.g., Cutler et al., 2004, 2008; García Lecumberri & Cooke, 2006; Jin & Liu, 2014; Mayo et al., 1997; Mi et al., 2013) as a masker, while only a few employed white noise (e.g., Bent & Bradlow, 2003; Bradlow & Bent, 2002; Shimizu et al., 2002). However, it has been shown that different kinds of maskers affect speech perception in different ways (García Lecumberri & Cooke, 2006; Parikh & Loizou, 2005; Taitelbaum-Swead & Fostick, 2016; Van Engen, 2012; Zhang et al., 2021), and that the identification of particular phonemes can vary depending on the employed masker (Broersma & Scharenborg, 2010).

White noise has a flat spectral slope which means that all frequencies are masked equally. Humans do not perceive all frequencies equally, though. Instead, they hear frequencies in octaves, thus on a logarithmic, not a linear scale, which means that the higher the frequency is, the louder it will be perceived. So even though white noise technically masks all frequencies to the same degree, the masking of higher frequencies will be perceived as louder than that of lower frequencies. This, in turn, naturally leads to a stronger masking effect of F2 than of F1. As this study focuses on the perception of vowels along the F2 dimension and how the non-native vowels /y/ and /ø/, which have intermediate F2 values between the front unrounded and back rounded vowels, are categorized in quiet compared with adverse listening conditions, we chose white noise as a masker in this study.

### 2.3 Procedure

In each trial of the Oddity Discrimination Task, participants heard three speakers uttering a CVC pseudoword with an interstimulus interval of 1,000 ms. Each of the speakers was associated with a distinct image of a robot. The order of both the voices and pictures was held constant. Participants were instructed to find the odd one among the three speakers, that is, the one that produced a different word than the other two. Each speaker was connected to a different button on a regular keyboard. If participants did not hear a difference between the speakers, they were supposed to press the spacebar. If no key was pressed within 6 s after stimulus presentation, the next trial was presented.

There were three kinds of trials: test trials, control trials, and same trials. In test trials, only vowels of the same height were contrasted with each other. In control trials, high and mid vowels were contrasted with /aː/. Because /aː/ is acoustically the farthest away from those vowels and was thus hypothesized to be relatively easy to distinguish even in noise, this contrast served as a baseline for each listening condition. In the same trials, the three items were identical. Because these same trials only served as fillers to give participants the opportunity to indicate that they did not hear a difference between items even if there was one, they were not included in the analysis.

In test and control trials, the odd speaker, that is, the one differing from the other two, could be any of the three speakers. Hence, there were six possible orders of presentation for each trial: AAB, ABA, BAA, BBA, BAB, and ABB. Eight quadruple sets with six contrasts for each (e.g., for the high vowels: /iː/-/yː/, /yː/-/uː/, and /iː/-/uː/ in the test trials; /iː/-/aː/, /yː/-/aː/, and /uː/-/aː/ in the control trials) in six orders of presentation yielded 288 control and test trials. In addition, there was one same trial for each vowel of each quadruple set, resulting in 32 same trials. To avoid that every participant is tested on a total of 320 trials, two stimuli blocks were created as follows: Set 1 consisted of AAB, ABA, and BAA trials of half of the contrasts and BBA, BAB, and ABB of the other half. The order was then reversed for Set 2. For instance, for the quadruple of /pVm/, Set 1 tested /iː/-/yː/, /yː/-/uː/, and /yː/-/aː/ in the order AAB, ABA, and BAA, and /iː/-/uː/, /iː/-/aː/, and /uː/-/aː/ in the order BBA, BAB and ABB. The opposite distribution was then held for Set 2. The 32 same trials were the same in both sets. An example of the counterbalanced stimulus distribution is presented in the Appendix.

Every participant was assigned to one of the two sets and then tested on 176 trials (72 test trials, 72 control trials, and 32 same trials). These 176 trials were presented in random order and divided into three blocks between which participants could take a short break. Randomization took place offline, so the order of presentation was the same for all participants within the same test group.

In the noise condition, all stimuli in a trial had the same SNR. For each participant, half of the trials were presented in masking at SNR 8 dB, and half at SNR 0 dB. To counterbalance the occurrence of the two SNRs, two noise groups were created (Noise-A and Noise-B). For instance, the quadruple of /pVm/ was tested at SNR 8 dB in Noise-A, but at SNR 0 dB in Noise-B. Another quadruple such as /kVt/, however, was tested at SNR 0 dB in Noise-A, but at SNR 8 dB in Noise-B. Because the two noise groups were also divided into two separate sets, there were six test groups in total that participants were randomly assigned to: Quiet-1, Quiet-2, Noise-A1, Noise-A2, Noise-B1, and Noise-B2. Thus, the participants within any particular test group were exposed to the same set of stimuli in the same randomized order at the same noise level.

The study was run online with PsychoPy3 (Peirce et al., 2019) via the platform Pavlovia (www.pavlovia.org).

### 2.4 Data analysis

A total of 15,840 data points were collected (90 participants × 176 trials per participant); 2% of trials were left unanswered and were not considered in the analysis. The response variable was the accuracy of the participants’ answers. Because 17 participants of the English group reported that they had learned French, a language with both /y/ and /ø/ phonemes, the French learners and non-learners were first analyzed separately. However, because there were no significant differences between the two—neither in the test nor in the control trials nor in any of the listening conditions—they were conflated into a single group and analyzed together.

The data was analyzed with generalized linear mixed effects models in R (R Core Team, 2021), using the packages lme4 (Bates et al., 2015) and languageR (Baayen, 2008).

In the first step, we ran a number of likelihood ratio tests to test for the main effects of our fixed factors (Vowel contrast, Noise level, and Language group) and their interactions. For this, all factors were defined using sum coding, and significance was assessed by comparing nested models with each other (see Levy, 2018).

In the second step, we analyzed the simple effects of the English and German groups separately in logistic regression models, followed by post hoc pairwise comparisons of the various vowel contrasts of the test trials at each noise level. These pairwise comparisons were run using the emmeans package (Lenth, 2023). All comparisons were Tukey adjusted. In this analysis of the simple effects, all factors were treatment-coded.

All models contained random intercepts for Participant and Item. If a model did not converge, we changed the optimizer to “BOBYQA” or, if necessary, a different optimizer until convergence was reached. Random slopes could not be fitted for all models due to persistent convergence issues that could not be fixed by changing the optimizer or manually reducing the random effects structure (see Winter, 2020). Thus, we kept the random effects structure constant in the likelihood ratio tests without random slopes but used the maximally converging random effects structure when testing for simple effects, which will be specified for each model separately.

## 3 Results

First, we tested for main effects by comparing a model of our response variable (accuracy) as predicted by each of the three predictors (Vowel contrast, Noise level, and Language group) separately to a simpler model that excluded the particular predictor using a likelihood ratio test. The simple model thus only included the random effects structure. Comparing this simpler model to a model including Vowel contrast as a predictor variable revealed a significant difference, χ^2^ (11) = 1924.2, *p* < .001, suggesting that not all vowel contrasts were equally easy to distinguish. Likewise, we found a significant effect for the predictors Noise level, χ^2^ (2) = 543.13, *p* < .001, and Language group, χ^2^ (1) = 9.5165, *p* = .002. In a next step, we compared a model predicted by Noise level and Language group to a model that included an interaction of both of these factors. The difference turned out to be significant, χ^2^ (2) = 14.614, *p* < .001, suggesting that accuracy in the different listening conditions varied for native German and native English speakers. We also found a significant difference between a model including an interaction of Noise level and Vowel contrast, and a simpler model without the interaction term, χ^2^ (22) = 455.21, *p* < .001, indicating that not all contrasts were equally affected by the noise masker. Finally, we compared a model predicted by Vowel contrast and Language group to a model that included an interaction of these factors. This comparison also turned out to be significant, χ^2^ (11) = 60.592, *p* < .001, which suggests that German and English speakers were not equally good at discriminating different vowel contrasts.

In the following subchapters, we analyze the English and German groups separately regarding their performance on specific vowel contrasts per noise level.

### 3.1 English group

**Figure 1.**
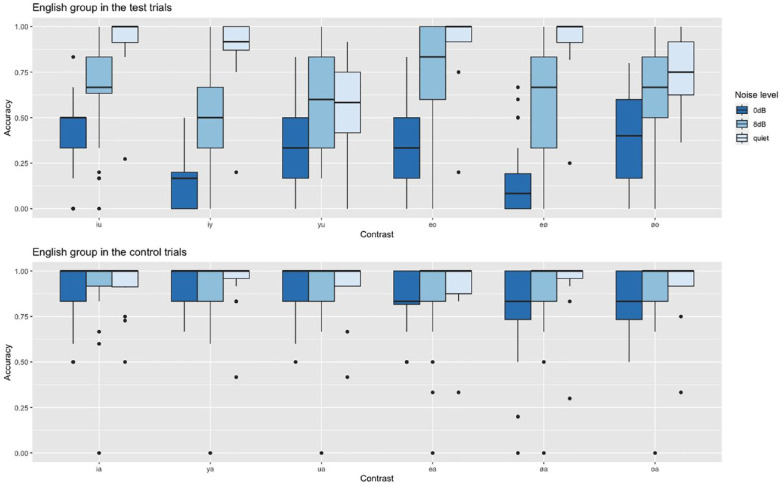
Native English speakers’ accuracy in the test and control trials in the three listening conditions.

First, we compared the native English speakers’ accuracy in test trials versus control trials. For this, we created a model including Trial type and Noise level as fixed effects and a random slope for Trial type by Participant. As the interaction between these two factors was significant (see Table 1), we ran three subsequent models for each noise level separately, each with a random slope for Trial type by Participant, to confirm that, as expected, performance on control trials was significantly better than on test trials in all listening conditions (quiet: β = −1.54, *SE* = 0.28, *z* = −5.4, *p* < .001; SNR 8 dB: β = −2.22, *SE* = 0.27, *z* = −8.23, *p* < .001; SNR 0 dB: β = −2.94, *SE* = 0.2, *z* = −14.99, *p* < .001). Because control trials with /a/ were not the focus of this study, they will not be analyzed in more detail. For a visualization of the results of each of the contrasts in test and control trials, see Figure 1.

**Table 1.** Results of the Mixed Effects Logistic Regression Model of the English Group, Analyzing the Effect of Trial Type and Noise Level on Accuracy.

Fixed effects	Estimate	*SE*	*z*-value	Pr(>|*z*|)
(Intercept: trialtypecontrol: noiselevel0 dB)	1.95	0.25	7.71	1.22e-14
trialtypetest	−3.01	0.2	−15.36	<2e-16
noiselevel8 dB	0.52	0.14	3.82	0.0001
noiselevelquiet	1.24	0.41	3	0.003
trialtypetest: noiselevel8 dB	1.01	0.17	6.03	1.67e-09
trialtypetest: noiselevelquiet	1.46	0.35	4.17	3.11e-05

Then, we ran a model looking at English speakers’ performance on only the test trials with Vowel contrast and Noise level as fixed effects (see Table 2). Results indicate that accuracy significantly decreased with higher noise levels. All contrasts were significantly easier to distinguish in quiet. Next, we run post hoc pairwise comparisons to compare the accuracy of each vowel contrast separately at each noise level. Note that random slopes could not be fitted in these models.

**Table 2.** Results of the Mixed Effects Logistic Regression Model of the English Group, Analyzing the Effect of Contrast and Noise Level on Accuracy in the Test Trials.

Fixed effects	Estimate	*SE*	*z*-value	Pr(>|*z*|)
(Intercept: contrastyu: noiselevel0 dB)	−0.92	0.33	−2.78	0.005
contrasteo	0.17	0.41	0.43	0.67
contrasteø	−1.05	0.43	−2.43	0.02
contrastiu	0.39	0.24	1.64	0.1
contrastiy	−1.25	0.28	−4.47	7.78e-06
contrastøo	0.2	0.41	0.49	0.62
noiselevel8 dB	1.25	0.24	5.31	1.09e-07
noiselevelquiet	1.1	0.37	2.99	0.003
contrasteo: noiselevel8 dB	0.68	0.34	2.01	0.04
contrasteø: noiselevel8 dB	1.04	0.36	2.93	0.003
contrastiu: noiselevel8 dB	0.02	0.33	0.07	0.95
contrastiy: noiselevel8 dB	0.89	0.36	2.47	0.01
contrastøo: noiselevel8 dB	−0.06	0.33	−0.17	0.87
contrasteo: noiselevelquiet	2.31	0.41	5.66	1.50e-08
contrasteø: noiselevelquiet	3.38	0.42	8	1.24e-15
contrastiu: noiselevelquiet	2.14	0.41	5.28	1.33e-07
contrastiy: noiselevelquiet	3.43	0.42	8.23	<2e-16
contrastøo: noiselevelquiet	0.88	0.35	2.55	0.01

#### 3.1.1 Quiet

Apart from the /y/-/u/ and /ø/-/o/ contrasts, the English group answered more than 88% of all test contrasts correctly (see Figure 1). For /y/-/u/, response accuracy was 57% only, which was significantly lower than all other contrasts (see Table 3). Furthermore, the contrast /ø/-/o/ proved to be rather difficult as well as accuracy was 75%, which was significantly lower than all other contrasts except compared with /i/-/y/ and /y/-/u/. No other differences reached significance.

**Table 3.** Pairwise Comparisons of English Speakers’ Accuracy on Test Contrasts in Quiet Listening Conditions.

Contrast	Estimate	*SE*	df	*z*-ratio	*p*-value
yu–eo	−2.67	0.43	Inf	−6.15	<0.0001
yu–eø	−2.68	0.43	Inf	−6.19	<0.0001
yu–iu	−2.8	0.37	Inf	−7.55	<0.0001
yu–iy	−2.18	0.32	Inf	−6.85	<0.0001
yu–øo	−1.07	0.36	Inf	−3.02	0.03
eo–eø	−0.009	0.43	Inf	−0.02	1
eo–iu	−0.13	0.5	Inf	−0.26	1
eo–iy	0.49	0.47	Inf	1.03	0.91
eo–øo	1.6	0.37	Inf	4.37	0.0002
eø–iu	−0.12	0.5	Inf	−0.24	1
eø–iy	0.5	0.47	Inf	1.06	0.9
eø–øo	1.6	0.36	Inf	4.41	0.0001
iu–iy	0.62	0.41	Inf	1.5	0.67
iu–øo	1.73	0.45	Inf	3.83	0.002
iy–øo	1.11	0.41	Inf	2.7	0.08

#### 3.1.2 SNR 8 dB

Although the contrasts between the rounded vowels /y/-/u/ and /ø/-/o/ were the most challenging in quiet, this effect disappeared once noise was added. Here, the accuracy for the contrasts between the front vowels decreased: /i/-/y/ (accuracy = 51%) and /e/-/ø/ (accuracy = 59%). Accuracy for the former was significantly lower than for /i/-/u/ trials, and accuracy for the latter was significantly lower than for /e/-/o/ trials (see Table 4).

**Table 4.** Pairwise Comparisons of English Speakers’ Accuracy on Test Contrasts at SNR 8 dB.

Contrast	Estimate	*SE*	df	*z*-ratio	*p*-value
yu**–**eo	−0.94	0.52	Inf	−1.81	0.46
yu**–**eø	−0.002	0.51	Inf	0.004	1
yu**–**iu	−0.59	0.25	Inf	−2.34	0.18
yu**–**iy	0.4	0.24	Inf	1.7	0.53
yu–øo	−0.26	0.51	Inf	−0.51	1
eo**–**eø	0.94	0.25	Inf	3.78	0.002
eo**–**iu	0.35	0.52	Inf	0.68	0.98
eo**–**iy	1.34	0.52	Inf	2.59	0.1
eo–øo	0.68	0.25	Inf	2.69	0.08
eø**–**iu	−0.58	0.52	Inf	−1.13	0.87
eø**–**iy	0.41	0.51	Inf	0.8	0.97
eø–øo	−0.26	0.24	Inf	−1.12	0.87
iu**–**iy	0.99	0.25	Inf	3.96	0.001
iu–øo	0.32	0.52	Inf	0.62	0.99
iy**–**øo	−0.67	0.51	Inf	−1.31	0.78

#### 3.1.3 SNR 0 dB

We see that the perceptual shift that we observed at SNR 8 dB increased further at an SNR of 0 dB. Accuracy for /i/-/y/ trials dropped to 14%, which was significantly lower than the accuracy for /i/-/u/ (42%), /y/-/u/ (34%), /e/-/o/ (36%), and /ø/-/o/ trials (38%) (see Table 5). Similarly, the other front vowel contrast /e/-/ø/ (accuracy = 15%) was significantly more difficult than both of the other mid vowel contrasts /e/-/o/ and /ø/-/o/, and than /i/-/u/. No other differences reached significance.

**Table 5.** Pairwise Comparisons of English Speakers’ Accuracy on Test Contrasts at SNR 0 dB.

Contrast	Estimate	*SE*	df	*z*-ratio	*p*-value
yu–eo	−0.14	0.45	Inf	−0.31	1
yu–eø	1.08	0.47	Inf	2.3	0.19
yu–iu	−0.36	0.23	Inf	−1.56	0.63
yu–iy	1.26	0.28	Inf	4.52	0.0001
yu–øo	−0.22	0.45	Inf	−0.48	1
eo–eø	1.22	0.27	Inf	4.5	0.0001
eo–iu	−0.22	0.44	Inf	−0.51	1
eo–iy	1.39	0.47	Inf	2.96	0.04
eo–øo	−0.08	0.23	Inf	−0.34	1
eø–iu	−1.44	0.47	Inf	−3.09	0.02
eø–iy	0.18	0.49	Inf	0.37	1
eø–øo	−1.29	0.27	Inf	−4.77	<0.0001
iu–iy	1.62	0.28	Inf	5.88	<0.0001
iu–øo	0.15	0.44	Inf	0.33	1
iy–øo	−1.47	0.47	Inf	−3.12	0.02

### 3.2 German group

**Figure 2.**
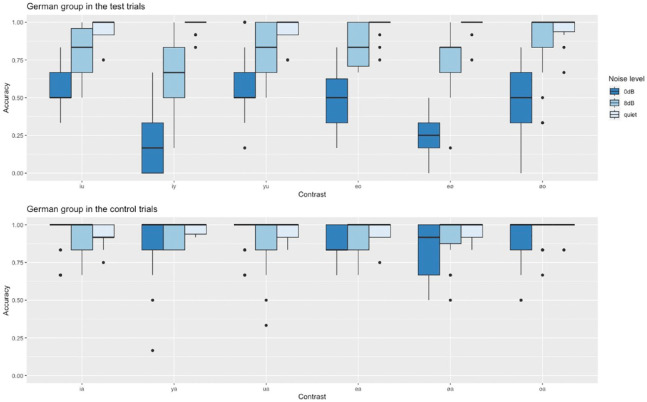
Native German speakers’ accuracy in the test and control trials in the three listening conditions.

As for the English group, we first compared German speakers’ accuracy in test trials versus control trials and thus created a model including Trial type and Noise level as fixed effects and random slopes for Trial type by Participant and Noise level by Item. As the interaction between these two factors turned out to be significant (see Table 6), we ran three additional models for each noise level separately (each including random slopes for Trial type by Participant) to test whether the German group, like the English group, performed significantly better on test trials than on control trials. This was only the case for the two noise conditions, but not for the quiet listening condition (quiet: β = 0.51, *SE* = 0.39, *z* = 1.3, *p* = .2; SNR 8 dB: β = −1.55, *SE* = 0.21, *z* = −7.41, *p* < .01; SNR 0 dB: β = −2.78, *SE* = 0.21, *z* = −12.95, *p* < .01). Control trials will not be discussed any further as they were not the main focus of this study. For a visualization of the German group’s results on test and control trials, however, see Figure 2.

**Table 6.** Results of the Mixed Effects Logistic Regression Model of the German Group, Analyzing the Effect of Trial Type and Noise Level on Accuracy.

Fixed effects	Estimate	*SE*	*z*-value	Pr(>|*z*|)
(Intercept: trialtypecontrol: noiselevel0 dB)	2.49	0.21	11.72	<2e-16
trialtypetest	−2.83	0.19	−14.74	<2e-16
noiselevel8 dB	0.48	0.22	2.19	0.03
noiselevelquiet	0.88	0.33	2.67	0.008
trialtypetest: noiselevel8 dB	1.33	0.19	6.98	2.95e-12
trialtypetest: noiselevelquiet	2.85	0.37	7.75	9.34e-15

In another model that included Vowel contrast and Noise level as fixed effects, we examined German speakers’ performance on only the test trials in more detail. Results suggest that while accuracy decreased overall with rising noise levels, discriminating the front vowels /i/-/y/ and /e/-/ø/ became particularly difficult in noise as we had previously observed for the English group (see Table 7). Note that random slopes could not be fitted in this model.

**Table 7.** Results of the Mixed Effects Logistic Regression Model of the German Group, Analyzing the Effect of Contrast and Noise Level on Accuracy in the Test Trials.

Fixed effects	Estimate	*SE*	*z*-value	Pr(>|*z*|)
(Intercept: contrastyu: noiselevel0 dB)	0.52	0.33	1.58	0.11
contrasteo	−0.62	0.44	−1.4	0.16
contrasteø	−1.81	0.45	−4.02	5.81e-05
contrastiu	−0.14	0.23	−0.61	0.54
contrastiy	−2.08	0.26	−7.97	1.51e-15
contrastøo	−0.61	0.44	−1.38	0.17
noiselevel8 dB	1.43	0.28	5.14	2.74e-07
noiselevelquiet	2.74	0.44	6.35	2.21e-10
contrasteo: noiselevel8 dB	0.64	0.39	1.63	0.1
contrasteø: noiselevel8 dB	1.25	0.38	3.27	0.001
contrastiu: noiselevel8 dB	−0.17	0.38	−0.45	0.66
contrastiy: noiselevel8 dB	0.88	0.38	2.32	0.02
contrastøo: noiselevel8 dB	0.86	0.4	2.16	0.03
contrasteo: noiselevelquiet	1.33	0.65	2.04	0.04
contrasteø: noiselevelquiet	2.54	0.66	3.84	0.0001
contrastiu: noiselevelquiet	0.43	0.57	0.76	0.45
contrastiy: noiselevelquiet	3.27	0.72	4.51	6.52e-06
contrastøo: noiselevelquiet	0.6	0.55	1.08	0.28

#### 3.2.1 Quiet

As expected, German participants performed at ceiling, hence the post hoc pairwise comparisons revealed no significant differences between any contrasts in quiet listening conditions (see Table 8).

**Table 8.** Pairwise Comparisons of German Speakers’ Accuracy on Test Contrasts in Quiet Listening Conditions.

Contrast	Estimate	*SE*	df	*z*-ratio	*p*-value
yu–eo	−0.89	0.63	Inf	−1.42	0.72
yu–eø	−0.9	0.63	Inf	−1.43	0.71
yu–iu	−0.29	0.54	Inf	0.54	0.99
yu–iy	−1.2	0.69	Inf	−1.74	0.51
yu–øo	−0.14	0.52	Inf	−0.27	1
eo–eø	−0.01	0.73	Inf	−0.01	1
eo–iu	0.6	0.65	Inf	0.92	0.94
eo–iy	−0.31	0.79	Inf	−0.4	1
eo–øo	0.75	0.64	Inf	1.17	0.85
eø–iu	0.61	0.65	Inf	0.93	0.94
eø–iy	−0.3	0.79	Inf	−0.38	1
eø–øo	0.76	0.64	Inf	1.19	0.84
iu–iy	−0.91	0.71	Inf	−1.28	0.8
iu–øo	0.15	0.55	Inf	0.27	1
iy–øo	1.06	0.7	Inf	1.51	0.66

#### 3.2.2 SNR 8 dB

At an SNR of 8 dB, the first perceptual difficulties emerged for the native German speakers (see Table 9). Accuracy is still relatively high, with means ranging from 80% to 90%, with one exception, however: /i/-/y/. Discriminating the high front vowels has become significantly more difficult than discriminating the other high vowels from each other (/i/-/u/: 81%; /y/-/u/: 85%; but /i/-/y/: 66%). No other differences reached significance.

**Table 9.** Pairwise Comparisons of German Speakers’ Accuracy on Test Contrasts at SNR 8 dB.

Contrast	Estimate	*SE*	df	*z*-ratio	*p*-value
yu–eo	−0.01	0.63	Inf	0.01	1
yu–eø	0.61	0.62	Inf	1	0.92
yu–iu	0.34	0.31	Inf	1.12	0.88
yu–iy	1.3	0.29	Inf	4.5	0.0001
yu–øo	−0.24	0.64	Inf	0.38	1
eo–eø	0.61	0.3	Inf	2.05	0.31
eo–iu	0.33	0.62	Inf	0.53	0.99
eo–iy	1.29	0.61	Inf	2.11	0.28
eo–øo	−0.25	0.33	Inf	−0.75	0.98
eø–iu	−0.27	0.61	Inf	−0.45	1
eø–iy	0.68	0.6	Inf	1.14	0.87
eø–øo	−0.85	0.31	Inf	−2.77	0.06
iu–iy	0.96	0.27	Inf	3.51	0.006
iu–øo	−0.58	0.63	Inf	−0.92	0.94
iy–øo	−1.53	0.62	Inf	−2.48	0.13

#### 3.2.3 SNR 0 dB

The difficulty to differentiate the high front vowels /i/ and /y/ that we observed at SNR 8 dB solidified at an SNR of 0 dB as this contrast has become significantly more difficult than all other contrasts except for /e/-/ø/ (see Table 10). Performance on /e/-/ø/ trials was significantly lower than all other contrasts as well. Accuracy on these two front vowel contrasts dropped to a mere 20% for /i/-/y/ and 23% for /e/-/ø/.

**Table 10.** Pairwise Comparisons of German Speakers’ Accuracy on Test Contrasts at SNR 0 dB.

Contrast	Estimate	*SE*	df	*z*-ratio	*p*-value
yu–eo	0.58	0.39	Inf	1.48	0.68
yu–eø	1.75	0.41	Inf	4.33	0.0002
yu–iu	0.14	0.23	Inf	0.61	0.99
yu–iy	2.0	0.26	Inf	7.79	<0.0001
yu–øo	0.57	0.39	Inf	1.46	0.69
eo–eø	1.17	0.24	Inf	4.91	<0.0001
eo–iu	−0.44	0.39	Inf	−1.13	0.87
eo–iy	1.42	0.41	Inf	3.49	0.006
eo–øo	−0.01	0.22	Inf	−0.4	1.0
eø–iu	−1.61	0.4	Inf	−4.0	0.001
eø–iy	0.25	0.42	Inf	0.6	0.99
eø–øo	−1.18	0.24	Inf	−4.94	<0.0001
iu–iy	1.86	0.26	Inf	7.3	<0.0001
iu–øo	0.43	0.39	Inf	1.11	0.88
iy–øo	−1.43	0.41	Inf	−3.51	0.01

## 4 Discussion

In this study, we examined English and German speakers’ perception of German vowel contrasts in quiet and white noise at SNRs of 8 and 0 dB in an Oddity Discrimination Task. The front rounded vowels /y/ and /ø/, which are not part of the English vowel inventory, were in focus. Previous studies have found that native speakers of English have difficulties in discriminating these vowels from back rounded vowels (e.g., Levy, 2009; Levy & Strange, 2008; Strange et al., 2005).

In quiet, we found that English speakers struggle to distinguish the front rounded and back rounded vowels. The /y/-/u/ contrast turned out to be the most challenging, which is in line with previous studies (Levy, 2009; Strange et al., 2005). English allows great variation for the production of /u/, which is typically fronted in alveolar contexts in many English varieties (Cox & Palethorpe, 2001; Hawkins & Midgley, 2005; Strange et al., 2005). Our results thus suggest that English speakers treat /y/ as an allophone of /u/.

Regarding the /ø/-/o/ contrast, Strange et al. (2005) argue that /ø/ should be discriminated from back rounded vowels rather easily because /ø/ could not be clearly assimilated to a single English category and was, therefore, deemed uncategorizable. This is not quite what we found in this study. Accuracy for the /ø/-/o/ contrast was above chance level, yet it was significantly lower than accuracy for all other contrasts, with the exception of /y/-/u/. Because our participants did not all speak the same variety of English, it is possible that some of them, such as the ones from England or Scotland, mapped the German /ø/ onto the mid-central vowel /ɜ/ that occurs in their varieties, but not in American English, for instance. Therefore, the /ø/-/o/ contrast might have been easier to perceive for some participants (i.e., speakers of British varieties) than for others.

When the signal was masked by white noise, however, there was a perceptual shift along the F2 dimension. As the noise level increased, it was no longer the rounded vowels that were the most difficult to discriminate, but the front vowels /i/-/y/ and /e/-/ø/. At an SNR of 0 dB, accuracy even dropped below chance level. These results were much stronger than anticipated but can be explained by the nature of the masker. In contrast to other kinds of noise, such as multitalker babble or speech-shaped noise, white noise is a particularly difficult masker (Taitelbaum-Swead & Fostick, 2016). Furthermore, formants are affected to different degrees in the presence of noise. Parikh and Loizou (2005) found that listeners must mainly rely on the transmitted F1 information when identifying vowels in noise because F2 is only partially detectable. In their study, it was therefore the vowels with similar F1 but different F2 values that were confused with each other, both in babble noise and in speech-shaped noise. This is in line with our finding for vowels masked by white noise. The F1 information on test trials was always very similar because only vowels of the same height were contrasted with each other, so participants had to rely on the deteriorated F2 information alone to identify the vowels. Although the difference in F2 between the front and back vowels was large enough to be detectable at least around 40% of the time at SNR 0 dB, the F2 values of the front unrounded and front rounded vowels were too similar to be detectable in white noise. The findings suggest that the phonological categorization difficulty in quiet turned into an acoustic challenge in noise. In the control trials with /a/, accuracy remained high even at 0 dB. For the contrasts involving /a/, it seems that the difference in the F1 dimension facilitated vowel recognition.

Interestingly, we found the same pattern for the German control group. Although accuracy was at ceiling in quiet, as expected, German speakers also showed immense difficulty in discriminating the front unrounded from the front rounded vowels as the noise level increased. However, they were less affected by noise than the English group. This suggests that even though pseudo-words were used—and hence neither group could use lexical information to recover from the disruption in the speech signal—there was still a clear native advantage. That means, German speakers were able to exploit some low-level phonetic cues that were not available for the naïve non-native group. This finding is in line with previous studies such as García Lecumberri and Cooke (2006) and Cutler et al. (2008). Furthermore, the native advantage in speech perception in adverse listening conditions does not only pertain to high-level word identification tasks (e.g., Mayo et al., 1997; Rogers et al., 2006) but also to low-level vowel discrimination tasks with pseudowords.

Finally, the accuracy rates of both the English and the German groups were surprisingly low at SNR 0 dB. Besides the stark masking effect of white noise (Taitelbaum-Swead & Fostick, 2016), the task demands were quite high in this study. Discriminating pseudowords is more difficult than discriminating real words. When speech is masked by noise, there is a high level of uncertainty, so the listener must deploy strategies to compensate for the distorted speech signal, such as using semantic context or probabilities based on lexical frequency. When listening to real words, listeners have been shown to be able to restore individual phonemes in the disrupted signal (Warren, 1970). But none of these strategies is possible when pseudowords are used, so the uncertainty remains. In addition, pseudowords demand more processing resources than words with lexical meaning (Wurm & Samuel, 1997). And finally, participants had to listen to three different speakers instead of just one. Hence, due to the high phonetic variability, participants could not rely on simple echoic recall but had to abstract the phonological information instead. It has been shown that talker variability leads to a decrease in perceptual performance compared with a single speaker (Mullennix et al., 1988) and that words spoken by a single speaker are recognized more quickly than words repeated by different speakers (Clapp et al., 2023). High task demands such as these lead to a high cognitive load, which can serve as an additional adverse condition in itself (Mattys & Wiget, 2011; for a review, see Mattys et al., 2012) and may have been another reason for the low accuracy scores, even for the native German group.

## 5 Conclusion

In sum, in line with previous studies, we found that native English speakers without any knowledge of German had difficulties in discriminating the German front rounded vowels /y/ and /ø/ from the back rounded vowels /u/ and /o/ in quiet. In white noise, however, there was a perceptual shift along the F2 dimension so that at an SNR of 0 dB, the difference between the front vowels /i/-/y/ and /e/-/ø/ was no longer perceivable, not even for the native German speakers. However, the German group was less affected by the noise distortion, which suggests a native advantage even in low-level discrimination tasks where no lexical information can help restore the speech signal.

## Appendix

### List of all stimuli

pVm: [piːm] – [pyːm] – [puːm] – [paːm]

vVp: [viːp] – [vyːp] – [vuːp] – [vaːp]

kVt: [kiːt] – [kyːt] – [kuːt] – [kaːt]

nVf: [niːf] – [nyːf] – [nuːf] – [naːf]

pVf: [peːf] – [pøːf] – [poːf] – [paːf]

fVk: [feːk] – [føːk] – [foːk] – [faːk]

dVt: [deːt] – [døːt] – [doːt] – [daːt]

nVp: [neːp] – [nøːp] – [noːp] – [naːp]

**Table A1.** Counterbalancing example across test groups.

Set	Contrast	Stimulus 1	Stimulus 2	Stimulus 3	Order	Test groups and SNR
**Set 1**	/iː/-/yː/	piːm	piːm	pyːm	AAB	Quiet-1, Noise-A1 at 8 dB, Noise-B1 at 0 dB
		piːm	pyːm	piːm	ABA	
		pyːm	piːm	piːm	BAA	
	/iː/-/uː/	piːm	puːm	puːm	ABB	
		puːm	piːm	puːm	BAB	
		puːm	puːm	piːm	BBA	
**Set 2**	/iː/-/yː/	piːm	pyːm	pyːm	ABB	Quiet-2, Noise-A2 at 8 dB, Noise-B2 at 0 dB
		pyːm	piːm	pyːm	BAB	
		pyːm	pyːm	piːm	BBA	
	/iː/-/uː/	piːm	piːm	puːm	AAB	
		piːm	puːm	piːm	ABA	
		puːm	piːm	piːm	BAA	

## Notes

1. Five other participants who had been tested were excluded from all further analyses because they had (some) knowledge of German (*n* = 2), or were bilingual (English-French and English-Chinese, *n* = 3). The exclusion of these bilinguals was necessary because at least one of the critical vowels of this study, /y/ and /ø/, occurs in these languages. Other bilingual (6 English-Spanish, 2 English-Urdu, 1 English-Scots, 1 English-Arabic, 1 English-Irish, and 1 English-Malay) and trilingual participants (1 English-Spanish-Italian) were kept for all further analyses. Note that some participants did not disclose their gender (*n* = 4) or their age (*n* = 7).

2. One additional participant had to be excluded from the analysis as she never pressed the spacebar, one of the four response keys, during the experiment. The three bilingual participants (1 German-Arabic, 1 German-Italian, and 1 German-Transylvanian Saxon) were included in the analysis. Note that some participants did not disclose their gender (*n* = 1) or their age (*n* = 6).

3. Due to the Covid restrictions during the pandemic, the sound-proof booth could not be used for stimuli recording.
